# Liposome bupivacaine for postsurgical pain in an obese woman with chronic pain undergoing laparoscopic gastrectomy: a case report

**DOI:** 10.1186/1752-1947-8-21

**Published:** 2014-01-22

**Authors:** Peter M Bertin

**Affiliations:** 1 Excela Health Westmoreland Hospital, 532 West Pittsburgh Street, Greensburg, PA 15601, USA

**Keywords:** Postsurgical analgesia, Laparoscopic sleeve gastrectomy, Liposome bupivacaine

## Abstract

**Introduction:**

To reduce incidence and severity of postsurgical pain and minimize the effect of its clinical and economic correlates, multimodal therapy for surgical patients is recommended. In this report, we discuss the use of liposome bupivacaine, a novel multivesicular formulation of bupivacaine indicated for single-dose infiltration into the surgical site to produce postsurgical analgesia, as part of a multimodal analgesic regimen in a patient with a history of chronic pain scheduled to undergo laparoscopic sleeve gastrectomy. To the best of our knowledge, this is the first published report of liposome bupivacaine in the setting of laparoscopic sleeve gastrectomy.

**Case presentation:**

A 35-year-old white woman with morbid obesity was admitted for laparoscopic sleeve gastrectomy to lose weight prior to hip replacement surgery. Because of a complicated medical history that included rheumatoid arthritis, fibromyalgia, diabetes mellitus, hypertension, and chronic pain, for which she was receiving high doses of opioid analgesics, postsurgical pain management was a concern and she was considered a candidate for multimodal analgesia. At initiation of surgery, 50mL of lidocaine and epinephrine was infiltrated around the port sites. At the conclusion, 25mL of normal sterile saline was added to a 20mL vial of liposome bupivacaine (266mg) and injected around the port sites and at the site of liver retraction. Laparoscopic sleeve gastrectomy was successfully completed. Our patient was discharged to the postanesthesia care unit for approximately four hours before discharge to the surgical floor with a pain score of 5 (11-point scale; 0 = no pain, 10 = worst possible pain). Her postoperative course was uneventful; no adverse events were recorded during surgery or during the remainder of her hospital stay. Our patient was discharged on the same opioid regimen used previously for control of her preexisting chronic pain.

**Conclusions:**

Liposome bupivacaine use in this morbidly obese patient undergoing laparoscopic sleeve gastrectomy provided analgesic efficacy and limited postsurgical opioids to a level comparable with her baseline opioid regimen for chronic pain. Given her complex medical history and previous issues with acute and chronic pain, we consider these results highly successful and continue to use liposome bupivacaine as part of a multimodal analgesic regimen in an effort to optimize postsurgical pain management.

## Introduction

Postsurgical pain is a potentially significant complication across a wide range of surgical procedures and has been found to contribute to increased risk of hospital readmission, increased per-patient costs [1], and decreased health-related quality of life in the immediate postsurgical period [2]. In an effort to reduce the incidence and severity of postsurgical pain, as well as the impact of its clinical and economic correlates, a task force established by the American Society of Anesthesiologists (ASA) has strongly endorsed multimodal therapy for surgical patients [3]. Multimodal analgesia involves the use of two or more drugs with differing mechanisms of action in an effort to maximize analgesic efficacy while reducing the risk and severity of adverse events (AEs) [3].

In this report, we discuss the use of liposome bupivacaine (EXPAREL®; Pacira Pharmaceuticals, Inc., Parsippany, NJ, USA), a novel multivesicular formulation of bupivacaine indicated for single-dose infiltration into the surgical site to produce postsurgical analgesia [4]. It was used as part of a multimodal analgesic regimen in a patient who had a history of chronic pain and was scheduled to undergo laparoscopic sleeve gastrectomy (LSG).

## Case presentation

**Figure 1:**
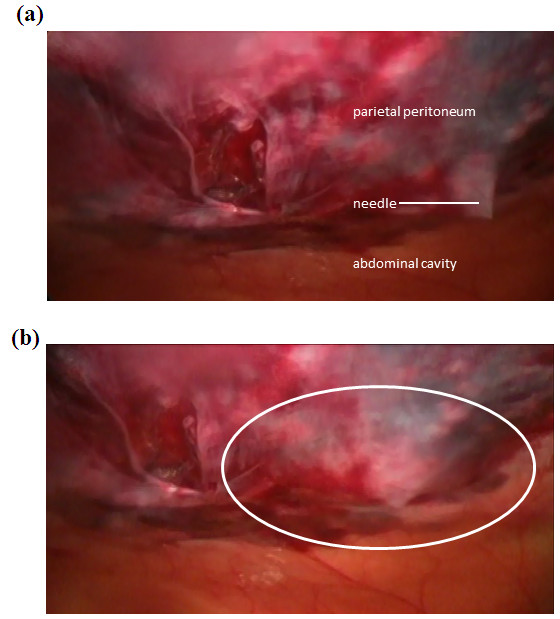
**Laparoscopic view of infiltration of liposome bupivacaine at 12-mm port site. (a)** Insertion of needle into deep tissue layers. Advancement of needle is stopped just prior to penetration of the parietal peritoneum. **(b)** Infiltration of liposome bupivacaine and dispersion of fluid to form a wheal (outlined by white oval). This process was repeated in four quadrants around the site of the trocar to form a field block. Photographs courtesy of Peter M. Bertin, DO.

A 35-year-old white woman with morbid obesity (height, 5 feet 1.5 inches; weight, 305 pounds; body mass index, 56kg/m^2^) and normal renal function (baseline creatinine, 0.6mg/dL) was admitted to our institution for LSG. She required weight loss in order to qualify for hip replacement surgery. Her past medical history was significant for rheumatoid arthritis, fibromyalgia, diabetes mellitus, hypertension, and obstructive sleep apnea. Our patient also had a history of chronic pain, for which she was receiving high doses of opioid analgesics (including transmucosal fentanyl, controlled-release oral morphine, and oral tramadol; see Table 1). The source of our patient’s pain was mostly in her joints, as a result of rheumatoid arthritis. In addition, our patient reported previously experiencing severe pain necessitating a prolonged hospital stay (>2 days) following a standard laparoscopic cholecystectomy. Given our patient’s history of both acute postsurgical and chronic pain and high baseline opioid usage, she was a desirable candidate for an opioid-reducing, multimodal pain management strategy.At the initiation of surgery prior to trocar insertion, 50mL of 1% lidocaine and epinephrine (1:100,000) was infiltrated around the port sites. At the conclusion of the procedure (which was more than 20 minutes after administration of the lidocaine), 25mL of 0.9% preservative-free normal sterile saline was added to a 20-mL vial of liposome bupivacaine (266mg) for a total volume of 45mL. It was injected using a spinal needle around the port sites (Figure 1): the flanking 5-mm port sites received 5mL each; the central 12-mm ports, 15mL each; and 5mL was infused at the site of liver retraction. The dose of liposome bupivacaine was based on the surgical site and the volume required to cover the area. Aided by visualization provided by the laparoscope, liposome bupivacaine was infiltrated directly into the deep tissue with the needle. The needle was slowly withdrawn so that infiltration of liposome bupivacaine was targeted primarily to the myofascial level. Our patient was administered a total of 925μg of IV fentanyl during surgery.

**Table 1:** Opioid use at baseline and after laparoscopic sleeve gastrectomy

**Day**	**Drug**	**Dosage × number of administrations/day**	**IV morphine equivalent per dose**	**Daily IV morphine equivalent dose per drug**	**Total daily IV morphine equivalent opioid dose**
**Presurgery baseline**					
NA	Transmucosal fentanyl citrate, oral	600μg × 4	15mg	60mg	
	Morphine CR, oral	30mg × 2	10mg	20mg	80mg (+ tramadol)
	Tramadol, oral	50mg as needed	NA^a^	NA^a^	
**Administered in the operating room**					
1	Fentanyl, IV	925μg × 1	92.5mg	92.5mg	
**Administered in the postanesthesia care unit**					
1	Hydromorphone, IV	0.5mg × 6	3.3mg	19.8mg	
**Administered during hospital stay**					164.1mg
1	Transmucosal fentanyl citrate, oral	200μg × 2	5mg	10mg	
	Morphine CR, oral	30mg × 1	10mg	10mg	
	Hydromorphone, IV	1mg × 4	6.7mg	26.8mg	
	Oxycodone + APAP, oral solution	10mg × 1	5mg	5mg	
2	Transmucosal fentanyl citrate, oral	600μg × 2	15mg	30mg	
	Morphine CR, oral	30mg × 2	10mg	20mg	
	Hydromorphone, IV	1mg × 3	6.7mg	20.1mg	90.1mg
	Oxycodone + APAP, oral solution	10mg × 3	5mg	20mg	
		5mg × 2	2.5mg		
3 (before discharge)	Transmucosal fentanyl citrate, oral	600μg × 3	15mg	45mg	
	Morphine CR, oral	30mg × 1	10mg	10mg	60mg
	Oxycodone + APAP, oral solution	10mg × 1	5mg	5mg	

^a^Conversion of oral tramadol to morphine equivalent not available. APAP, acetaminophen; CR, controlled-release; IV, intravenous; NA, not applicable.

LSG was successfully completed. Our patient was discharged to the postanesthesia care unit, where she stayed for approximately four hours before discharge to the surgical floor with a pain score of 5 (11-point scale; 0 = no pain, 10 = worst possible pain). The postoperative course was uneventful; no AEs were recorded during surgery or during the remainder of our patient’s hospital stay. Her pain scores ranged from 5 to 8 during her hospital stay; however, this was consistent with her pain before surgery, which was always ≥4 due to her history of chronic pain. She reported that the majority of her pain was musculoskeletal in location and related to her rheumatoid arthritis and fibromyalgia and not related to the surgical site. Analgesic medications administered to the patient are summarized in Table 1 and included intravenous (IV) hydromorphone, oral transmucosal fentanyl citrate, controlled-release morphine tablets, and oxycodone/acetaminophen oral solution. In order to facilitate comparisons, opioid doses have been converted to their IV morphine equivalents. Total opioid use (IV morphine equivalent) was 51.8mg on day 1 (after surgery), 90.1mg on day 2, and 60mg on day 3 (before discharge).

Our patient was discharged routinely two days after surgery, with the same home medication regimen as before surgery, along with a prescription for oxycodone/acetaminophen 5/325mg four times daily as needed.

## Discussion

Given our patient’s complicated medical history and experience with both acute postsurgical and chronic pain, there was a strong expectation of significant acute postsurgical pain after LSG, which would have normally required increased opioid use for adequate pain control. Opioid use is problematic in obese patients: calculation of adequate doses is difficult using traditional body surface area measurements, given the disparity between lean and total body mass; obese patients are particularly susceptible to opioid-induced respiratory depression; and opioid use may exacerbate obstructive sleep apnea in affected patients [5,6]. These are the issues that dissuaded us from the routine postsurgical use of patient-controlled analgesia, which, combined with inadequate supervision during hospitalization, can result in excessive opioid use and increased risk of AEs in obese patients.

These considerations supported the use of multimodal analgesia consistent with ASA recommendations; however, we remained concerned about the potential for adequate pain control given the duration of action of most local anesthetics (<10 hours) [7,8]. Therefore, we administered liposome bupivacaine, a multivesicular formulation of bupivacaine designed to allow diffusion of the drug over an extended period, around the surgical ports and at the site of liver retraction. In a pivotal soft tissue trial (hemorrhoidectomy), liposome bupivacaine was shown to provide postsurgical analgesia and reduce opioid use for 72 hours after surgery [4,9]. To the best of our knowledge, this is the first published report of a multimodal analgesic regimen involving liposome bupivacaine in the setting of LSG. It should be noted that in our practice, we now infiltrate liposome bupivacaine preemptively at the start of the procedure and no longer administer lidocaine plus epinephrine. Additionally, we now add 40mL of 0.9% preservative-free normal sterile saline to a 20-mL vial of liposome bupivacaine (266mg) for a total volume of 60mL for LSG procedures, in contrast to the total volume of 45mL used in the current report. In our experience, the increased volume results in greater dispersion of liposome bupivacaine and, thus, potentially improved nerve coverage.

Common AEs following administration of liposome bupivacaine include nausea, constipation, and vomiting. Common neurologic AEs include dizziness, headache, somnolence, hypoesthesia, and lethargy; common cardiac AEs include tachycardia and bradycardia [4]. Our patient’s postsurgical stay was not complicated by any liposome bupivacaine-related AEs, opioid-related AEs (for example, nausea, constipation, respiratory depression), or by inadequate pain control; the length of stay (two days) was consistent with expectations. The patient was discharged on the same opioid regimen used previously for control of her preexisting chronic pain. Notably, during her inpatient stay, her use of opioids between surgery and discharge (51.8mg, 90.1mg, and 60mg IV morphine equivalent on days 1, 2, and 3, respectively) was comparable to her baseline daily opioid usage (80mg IV morphine equivalent plus tramadol as needed), even during the time period (≥12 hours) when the efficacy of conventional local anesthetics would be expected to wane.

## Conclusions

The use of liposome bupivacaine in this patient with morbid obesity undergoing LSG provided analgesic efficacy and limited the postsurgical use of opioids to a level comparable with the patient’s baseline opioid regimen for chronic pain. In light of the patient’s complex medical history and previous issues with acute and chronic pain, we consider these results highly successful and continue to use liposome bupivacaine as part of a multimodal analgesic regimen in an effort to optimize postsurgical pain management.

### Patient’s perspective

Following the gastrectomy, I lost sufficient weight and was able to have a hip replacement surgery. After my hip replacement surgery, the doctors changed some of my chronic pain medications around and I had many issues with pain control. I was in the hospital for 20 days. Of the six major surgeries I have had in my life, the recovery from this gastrectomy was the most pain-free and fastest of all.

## Consent

Written informed consent was obtained from the patient for publication of this case report and any accompanying images. A copy of the written consent is available for review by the Editor-in-Chief of this journal.

## Competing interests

Dr. Bertin has participated as a speaker and consultant for Pacira Pharmaceuticals, Inc.
